# Design of Hybrid Polymer Nanofiber/Collagen Patches Releasing IGF and HGF to Promote Cardiac Regeneration

**DOI:** 10.3390/pharmaceutics14091854

**Published:** 2022-09-02

**Authors:** Eloise Kerignard, Audrey Bethry, Chloé Falcoz, Benjamin Nottelet, Coline Pinese

**Affiliations:** Polymers for Health and Biomaterials, Institute of Biomolecules Max Mousseron (IBMM), CNRS, ENSCM, University of Montpellier, 34090 Montpellier, France

**Keywords:** bioactive nanofibers, growth factors delivery, collagen association, cardiac regeneration, electrospinning, hybrid scaffolds

## Abstract

Cardiovascular diseases are the leading cause of death globally. Myocardial infarction in particular leads to a high rate of mortality, and in the case of survival, to a loss of myocardial functionality due to post-infarction necrosis. This functionality can be restored by cell therapy or biomaterial implantation, and the need for a rapid regeneration has led to the development of bioactive patches, in particular through the incorporation of growth factors (GF). In this work, we designed hybrid patches composed of polymer nanofibers loaded with HGF and IGF and associated with a collagen membrane. Among the different copolymers studied, the polymers and their porogens PLA-Pluronic-PLA + PEG and PCL + Pluronic were selected to encapsulate HGF and IGF. While 89 and 92% of IGF were released in 2 days, HGF was released up to 58% and 50% in 35 days from PLA-Pluronic-PLA + PEG and PCL + Pluronic nanofibers, respectively. We also compared two ways of association for the loaded nanofibers and the collagen membrane, namely a direct deposition of the nanofibers on a moisturized collagen membrane (wet association), or entrapment between collagen layers (sandwich association). The interfacial cohesion and the degradation properties of the patches were evaluated. We also show that the sandwich association decreases the burst release of HGF while increasing the release efficiency. Finally, we show that the patches are cytocompatible and that the presence of collagen and IGF promotes the proliferation of C2C12 myoblast cells for 11 days. Taken together, these results show that these hybrid patches are of interest for cardiac muscle regeneration.

## 1. Introduction

Cardiovascular disease is the leading cause of death worldwide. Myocardial infarction and coronary heart diseases were responsible for 7.4 million deaths in 2015, according to the World Health Organisation [1]. The necrosis caused by infarction cannot regenerate on its own due to the low regenerative capacity of the myocardium, which results in a loss of functionality of part of the myocardium. Currently, this functionality can be restored by replacing the damaged organ, notably by a heart transplant. Although effective, this solution poses the problem of rejection and organ availability. In situ regeneration of the damaged part of the myocardium is another option, in particular through cell therapy and regeneration from biomaterials. Cellular therapy by injection of stem cells involves the direct injection of new cells on the damaged site, which can remodel the defective part. The first results are promising [2], but this therapy is still very expensive and time-consuming [3]. In contrast, the biomaterial-based regeneration approach provides a support for recruiting neighboring cells, thereby promoting regeneration of the damaged myocardium. One attractive strategy illustrating this approach is the design of cardiac patches made of biomaterials whose mechanical, degradation, and tissue integration properties are adapted to the physiology and regeneration of cardiac tissue. Furthermore, the suturability of the scaffold to the cardiac tissue is an important criterion to facilitate surgical implantation. To meet these requirements, scaffolds composed of nanofibers are widely used as cardiac regeneration patches because the entanglement of the nanofibers promotes superior suturability compared to a hydrogel. In addition, nanofiber mats are also widely used because their structure mimics the myocardium extra cellular matrix. These nanofibrous patches are generally produced by electrospinning from polymers such as PLA, PCL, PU, or PLGA [4,5,6]. More advanced examples include nanofibers with cell-stimulating properties, such as conductive nanofibers that allow cardiomyocytes to beat synchronously in response to electrical stimulation [4,7,8], or nanofibers loaded with biomolecules to stimulate cardiomyocytes [9]. It has been shown that VEGF and FGF embedded in nanofibers have positive effects on the regulation of survival, growth, and migration of cardiomyocytes [10]. Furthermore, a favorable effect of hepatocyte growth factor (HGF) and insulin-like growth factor (IGF) on cardiomyocytes was shown when released from nanoparticles [9,11]. This research suggests that the encapsulation of HGF and IGF in patches composed of degradable nanofibers could be of interest to provide sustained bioactivity to the patches.

In addition, to promote the integration of the nanofibers into the tissue, it has been shown that the presence of collagen, naturally present in cardiac tissue, is efficient [7]. While researchers are seeking to incorporate collagen into nanofibers by creating nanofibers from a polymer-collagen mixture [12,13], it has also been shown that electrospinning, due to the passage through an electric field, denatures the molecule. This is why physical associations between collagen and nanofibers are considered as interesting directions for the elaboration of future cardiac patches. Scaffolds composed of PLGA nanofiber with gelatin films have shown improved adhesion of hMSCs [14]; however, very few degradable collagen-nanofiber patches have been reported in the literature so far [15].

In this study, we designed and prepared hybrid cardiac patches composed of IGF- and HGF-loaded degradable polymeric nanofibers associated with a collagen membrane. For this purpose, we synthesized different (co)polymers based on PLA and PCL in order to modulate the release kinetics of GFs. The nanofibers were then associated by different methods with a collagen membrane, and the impact of the association on GF release was evaluated. Finally, we studied the cytocompatibility and bioactivity of the nanofiber patch, demonstrating the interest of such a patch for cardiac regeneration.

## 2. Materials and Methods

### 2.1. Materials

D,L-lactide was purchased from Purac. Tin(II) 2-ethylhexanoate (Sn(Oct)_2_), phosphate buffer solution (PBS), Bovine serum albumin, heat shock fraction V (BSA), Pluronic^®^ F-127 (12,600 g·mol^−1^), Sodium hydroxide (40 g·mol^−1^), Polycaprolactone (PCL) (Mn 80,000), and dichloromethane (DCM) (puriss.p.a, ACS reagent, ISO ≥ 99.9%) were supplied by Sigma. L-lactide (L-LA), D,L-lactide (D,L-LA), and glycolide were purchased from Purac. Collagen membrane (CCC sterilized membrane) and collagen solution were kindly supplied by Viscofan Bioengineering. 2,2,2-trifluoroéthanol, 99+% (TFE) was purchased from Fisher Scientific and ethanol from Honeywell. Poly(ethylene glycol) (PEG) 10,000 g·mol^−1^ (ROTIPURAN^®^ Ph.Eur) was received from Roth Sochiel EURL. Pierce™ BCA Protein Assay Kit (23227) and BSA FITC were purchased from Thermo Fischer Scientific, ELISA kit from Anticorps-enligne.fr, and growth factors (HGF recombinant human: Cat#11343417; IGF recombinant murine: Cat#12343317) from Immunotools. PrestoBlue^TM^, Dulbecco’s Modified Eagle Medium (DMEM/F-12), Fetal Bovine Serum (FBS), penicillin, streptomycin, and glutamine were purchased from Invitrogen. BD Falcon™ Tissue Culture Polystyrene (TCPS) 24-well plates were purchased from Becton Dickinson and Viton^®^ O-rings from Radiospares.

### 2.2. Synthesis of Copolymers

PLA-Pluronic-PLA and PLGA-Pluronic-PLGA block copolymers were synthesized by ring opening polymerization following an already described procedure [16,17,18]. The amount of each monomer was varied to obtain the different polymers, and the monomer/initiator ratio was fixed to obtain the targeted molecular weights of 100,000 g·mol^−1^ (Table S1). Predetermined amounts of D,L-LA or/and L-LA or/and glycolide monomers, Pluronic as an initiator and Sn(Oct)_2_ as a catalyst (10% molar/initiator OH) were introduce into a flask. For PLA50, benzyl alcohol was used as the initiator. After vacuum-nitrogen purge cycles, the flask was sealed under vacuum and placed at 125 °C for 8 days under stirring. The polymers were solubilized in DCM and purified by precipitation in cold ethanol. Finally, the resulting precipitate was dried under vacuum. The polymers were obtained with an average yield of 92%. The polymers’ molecular weights were calculated from the ^1^H NMR spectra using the integration of the peaks corresponding to the protons of the initiator and the methyne proton of lactic unit.

PLA-Pluronic-PLA: ^1^H-NMR (400 MHz, CDCl3) δ (ppm) = 5.1 (m, 1H, CO-CHCH_3_); 4.3 (1H, m, CHCH_3_–OH); 3.6 (s, 4H, CH_2 -_CH_2_-O), 3.5 (m, 2H, CH(CH_3_)-CH_2_-O), 3.4 (m, 1H, CH(CH_3_)-CH_2_-O), 1.5 (m, 3H, CO-CH(CH_3_)), 1.1 (m, 3H, CH(CH_3_)-CH_2_-O).

PLGA-Pluronic-PLGA polymers: ^1^H-NMR (400 MHz, CDCl3) δ (ppm) = 5.1 (m, 1H, CO-CHCH_3_), 4.8 (m, 2H, CO-CH_2_-O), 3.6 (s, 4H, CH_2_-CH_2_-O), 3.5 (m, 2H, CH(CH_3_)-CH_2_-O), 3.4 (m, 1H, CH(CH_3_)-CH_2_-O), 1.5 (m, 3H, CO-CH(CH_3_)), 1.1 (m, 3H, CH(CH_3_)-CH_2_-O).

### 2.3. Characterization of Polymers

Number average molecular weight (Mn) and dispersity (Ð) of the polymers were determined by size exclusion chromatography (SEC) conducted on a Shimadzu LC-200 AD Prominence system, equipped with an RID-20A refractive index signal detector, a PLgel MIXED-C guard column (Agilent, 5 μm, 50 × 7.5 mm), and two PLgel MIXED-C columns (Agilent, 5 μm, 300 × 7.5 mm). Polymers were dissolved in THF (5 mg/mL), and solutions were filtered through a Millipore membrane before the injection of 100 μL into the system. All measurements were performed at 30 °C, and the mobile phase was THF at 1 mL/min flow and 30 °C. The number average molecular weight (Mn) and weight average molecular weight (Mw) were expressed according to a polystyrene calibration.

The ^1^H NMR measurements were performed at 300 MHz with an AMX300 Bruker spectrometer using deuterated chloroform as the solvent. The chemical signals are expressed in ppm with respect to the tetramethylsilane (TMS) signal used as an internal reference.

### 2.4. Preparation and Characterization of Nanofiber Scaffolds

A first series of BSA-loaded fibers was prepared for the initial screening and selection of polymers. Briefly, polymers were dissolved in TFE at different concentrations (Table S2) and porogens PEG or Pluronic were added at the concentration of 14% (*w*/*w*) and 29% (*w*/*w*), respectively. Then, 7 mg of BSA was loaded into 200 µL of polymer/porogen solution before being vigorously mixed and electrospun.

GF-loaded nanofibers were prepared with the two selected polymers (PCL + Pluronic and PLA-Pluronic-PLA + PEG) (Table S3). First, GF were stabilized with BSA at 1/100 or 1/10 molar ratio for IGF and HGF, respectively. Thereafter, 500 ng of IGF and 500 ng of HGF (for release study) or 1000 ng of IGF (for biological properties study) were introduced in 200 µL of polymers solution at an aqueous phase/organic phase ratio of 9% (*v*/*v*), and the resulting solution was mixed vigorously before being electrospun.

The electrospinning process was carried out at room temperature at a relative humidity of 20–40%. All the electrospinning parameters are detailed in Tables S2 and S3. A volume of 200µL of mixed solution was loaded in a syringe with a 21 Gauge needle and fixed to a pump (KDS-100, KS scientific). A positive voltage between 12 kV and 11 kV was applied to the polymer solution dispensed at 0.65 or 0.9 mL·h^−1^ for PCL + Pluronic and PLA-Pluronic-PLA + PEG, respectively. The protein-loaded nanofibers were collected on a flat aluminum foil of 5.2 × 5.2 cm, maintained at 15 cm of the needle. The electrospun mat was further cut into scaffolds of 1.3 × 1.3 cm that were dried vacuum overnight for complete elimination of the solvent.

The surface morphology of the electrospun nanofibers was analyzed with SEM (Phenom ProX) after gold sputter coating. The average diameter of the fibers was measured using Image J software (https://imagej.nih.gov/ij/download.html, accessed on 20 July 2022) by randomly selecting 60 fibers.

### 2.5. Quantification of BSA and GF Loading and Release

#### 2.5.1. In Vitro Release

BSA-loaded and GF-loaded nanofiber scaffolds weighing between 1 and 3 mg (1.3 × 1.3 cm) were incubated at 37 °C in 2 mL of PBS under shaking (100 rpm). At each time point, 1 mL of buffer was collected and replaced by an equal volume of fresh PBS. The amount of released BSA was determined by a Pierce BCA kit, following the manufacturer’s protocol and measuring the absorbance at 562 nm using a UV–vis spectrophotometer. The percentage of cumulated release of BSA was presented as mean ± standards deviation of three samples. The amount of growth factor was determined by an ELISA kit, following the manufacturer’s protocol and measuring absorbance at 450 nm.

#### 2.5.2. Encapsulation Efficiency

The experimental loading efficiency of BSA and GF was adapted from other protocols [19,20] that had been modified to ensure improved extraction. Briefly, BSA- and GF-loaded nanofiber scaffolds were placed under vacuum overnight to remove all solvent and were then dissolved in 1 mL of DCM. Thereafter, 2 mL of PBS was added and vigorously mixed for 1 min. Then, the same amount of ethanol was added. After shaking for 1 min and centrifugated at 1500 rpm for 4 min, the aqueous phase was collected (E1). Then, 2 mL of PBS and 2 mL of ethanol were added to the remaining organic phase and vigorously mixed for 1 min after being centrifugated at 1500 rpm for 4 min. The aqueous phase was collected (E2). This process (E1 and E2) was repeated one more time (E3 and E4). The phases collected after each extraction were analyzed by BCA or ELISA kit, as describe above, to quantify the BSA or GF. The total amount of BSA or GF extracted was obtained by adding the amount of BSA or GF found in each extract. The extraction efficiency of BSA or GF from the polymer solution was also determined. For this purpose, known amounts of BSA or GF were added to polymer films and extracted using the same extraction protocol. Finally, the encapsulation efficiency (EE) of BSA and/or GF was calculated by Equation (1):

(1) $$\%\mathrm{EE}=\frac{\mathrm{Total}\;\mathrm{amount}\;\mathrm{of}\;\mathrm{BSA}/\mathrm{GF}\;\mathrm{released}+\;\mathrm{Total}\;\mathrm{amount}\;\mathrm{of}\;\mathrm{BSA}/\mathrm{GF}\;\mathrm{extracted}}{\mathrm{Amount}\;\mathrm{of}\;\mathrm{BSA}/\mathrm{GF}\;\mathrm{theoretically}\;\mathrm{incorporated}}\;\times \;100$$

### 2.6. Association of the Nanofibrous Scaffold and Collagen Membranes

#### 2.6.1. Association Methods

Two methods were developed to associate collagen membranes and loaded nanofiber scaffolds. The first method, referred as the “sandwich association” method, associates the collagen membrane with the nanofibers thanks to a collagen solution. Briefly, 100 µL of collagen solution was deposited on 1.5 cm × 1.5 cm collagen membranes. Subsequently, the nanofibrous scaffold (1.3 cm × 1.3 cm) was placed in contact with the collagen solution before 100 µL was deposited again. The sandwich association was then freeze-dried overnight. The second method, referred as the “wet association” method, combines a collagen membrane (1.5 cm × 1.5 cm) humidified with water and a nanofiber scaffold (1.3 cm × 1.3 cm) placed directly on the moisturized collagen surface. After association, the construct was placed at 37 °C overnight to reinforce the interfacial interactions between the two layers.

#### 2.6.2. Mechanical Tests

To quantify the interaction between the nanofiber scaffold and the collagen membrane, lap-shear type experiments were performed using an Instron 2710-203 with a 50 N load cell capacity in water at 37 °C. The upper clamp held the collagen membrane while the lower clamp held the nanofibrous scaffold. The force required to separate the layers and the strain at failure were measured at a speed of 10 mm/min and reported as mean ± SD.

#### 2.6.3. Swelling Ratio

The in vitro swelling ratio of BSA-loaded scaffolds (5.2 × 5.2 cm) was measured using the mass change of samples after immersion in PBS for 5 min (n = 3). The swelling ratio was calculated from Equation (2):

Swelling ratio (%) = [Mw − Mi]/Mw × 100(2)

where M_i_ is the initial dry mass of the samples before immersion in water and M_w_ is the mass of the hydrated samples.

#### 2.6.4. In Vitro Degradation

BSA-loaded scaffolds (5.2 × 5.2 cm) were immersed in 8 mL of PBS at 37 °C under constant stirring. The samples were removed from PBS at different times up to 40 days, and sample remaining mass were measured following Equation (3):

Remaining mass (%) =1 − (Mi − Mx)/Mi × 100(3)

where M_i_ is the initial dry mass of the samples before immersion in water and Mx is the dry mass after x time in PBS.

### 2.7. Cytocompatibility

Two tests were performed, including the cytotoxicity of patch extracts as well as tests with cells in direct contact with the patches.

The in vitro cytocompatibility of patches was tested following EN ISO 10993-12 standards protocol (n = 3). BSA-loaded patches were decontaminated with UV-C irradiation (2-min, 80 W). Scaffolds and control materials (non-cytotoxic polyethylene was used as negative control, while cytotoxic zinc stabilized polyurethane was used as positive control) were immerged in x ml of DMEM and were placed at 37 °C under agitation over 72 h. Meanwhile, L929 cells were seeded at 10^4^ cells per well (96-well plate) and allowed to attach for 24 h at 37 °C and 5% CO_2_. Then, the medium in contact with the materials was extracted and 100 µL was placed in contact with cells for an additional 24 h. After that time, the number of viable cells was obtained by a CellTiter Glo assay, based on quantification of the present ATP, which represents metabolically active cells.

C2C12 myoblast proliferation on the nanofibers-collagen association samples was evaluated over 11 days. Scaffolds (n = 4) of 1.9 cm^2^ were UV decontaminated and fixed in 24-well plates (TCPS non-treated for cells culture) using silicone sinker. After 24 h of FBS starvation, 2 × 10^4^ C2C12 cells at P6 were seeded on different samples and incubated in 1% FBS medium at 37 °C and 5% CO_2_. The number of cells was assessed after 1, 7, and 11 days of contact with the scaffolds using a PrestoBue assay that evaluates the transformation of weakly-fluorescent blue resazurin into highly fluorescent red resorufin through the mitochondrial activity of the cell.

## 3. Results

### 3.1. Synthesis

PLA-based polymers were synthesized by ring opening polymerization using Pluronic F-127 or benzyl alcohol as an initiator and Sn(Oct)2 as a catalyst. Table 1 presents the synthesized polymers, their molecular weight, and dispersity, as characterized by ^1^H-NMR (Figure S1) and SEC analyses.

The copolymers molecular weights determined by ^1^H NMR from the ratio between the methyne proton of the initiator and the methyne proton of the lactic unit were close to the targeted values (100 kg·mol^−1^). The compositions for the PLGA copolymers were also in good agreement with the initial monomers feed. SEC analyses showed a monomodal population with dispersity between 1.4 and 2.0. The molecular weights measured by SEC analysis were lower than those calculated by ^1^H NMR because of the amphiphilic nature of the Pluronic copolymers, which lead to changes of the hydrodynamic volume and therefore of the retention time.

### 3.2. BSA Loading and Release from Nanofibers

#### 3.2.1. BSA Loading

**Figure 1:**
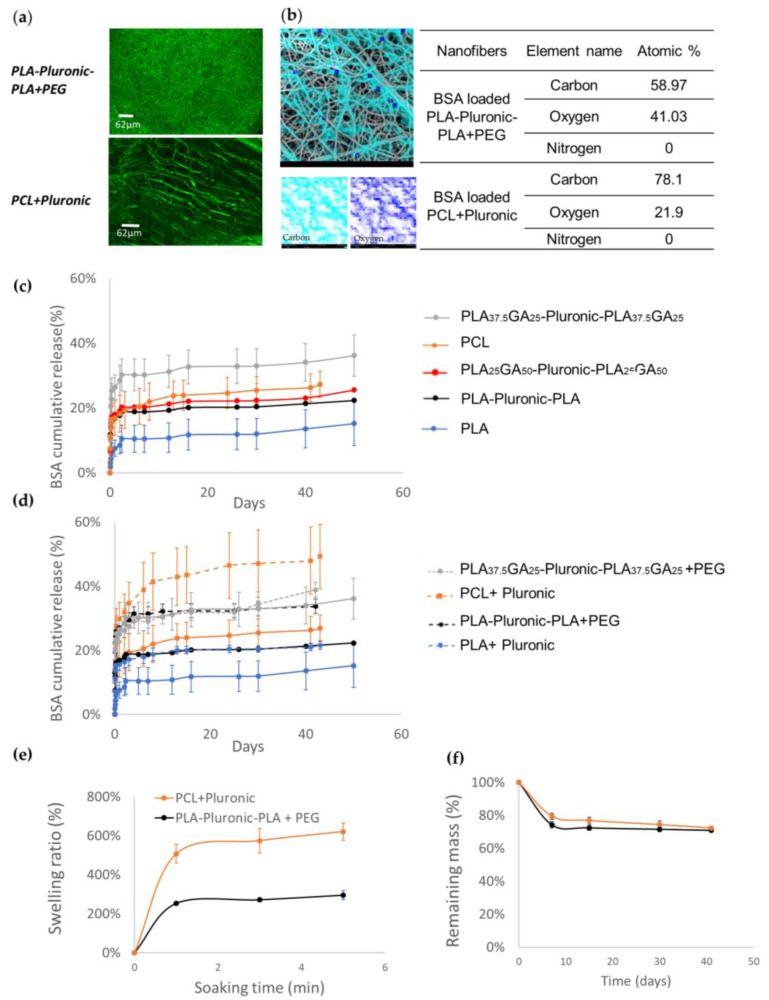
Selection of polymers based on the release kinetics of BSA: (**a**) Confocal images of the FITC-BSA-loaded PLA-Pluronic-PLA + PEG and PCL + Pluronic nanofibers that show homogenous BSA distribution; (**b**) SEM-EDX elemental map that show no nitrogen on the surface of PLA-Pluronic-PLA + PEG and PCL + Pluronic nanofibers, meaning that BSA is well encapsulated inside the nanofibers; (**c**) BSA release kinetics from polymeric nanofibers; (**d**) Increase of the release efficiency of BSA due to the addition of a porogen (9% of PEG or 5% of PluronicF127); (**e**) Swelling ratio of nanofiber scaffolds in PBS (n = 3); (**f**) In vitro degradation of nanofibers scaffolds in PBS (n = 3).

BSA is classically used to protect growth factors during the electrospinning process [21]. For this reason, we first evaluated the ability of the various polymers to encapsulate and release BSA. In order to visualize the BSA distribution into nanofibers, we incorporated FITC-BSA into PLA-Pluronic-PLA + PEG and PCL + Pluronic nanofibers. As shown on Figure 1a, BSA was homogenously distributed into nanofibers on their entire length.

The SEM-EDX analysis (Figure 1b) showed oxygen and carbon at the surfaces of the fibers, which was as expected considering the polymers used. However, no nitrogen of BSA was detected, which indicates that BSA was not on the surface of the nanofibers but was well encapsulated inside nanofibers. The same was observed for all BSA-loaded nanofibers, whatever the polymer used.

#### 3.2.2. BSA Release from Nanofibers

The release of BSA from the nanofibers composed of the different polymers was carried out to evaluate the release efficiency and to select the best polymers for further BSA/GF formulations. As shown in Figure 1c, all release profiles were similar, with an initial burst in the first 24 h followed by a very slow release over the whole release experiment. The nature of the polymers influenced the overall amount of BSA released.

In more details, PLA nanofibers led to the lowest BSA release, with only 15% in 50 days. The introduction of the hydrophilic Pluronic block in the polymer increased the release efficiency with PLA-Pluronic-PLA nanofibers releasing 22% over the same period. Finally, the introduction of glycolic acid units (GA) further enhanced the release, as demonstrated by the cumulative release values of 25% and 36% obtained for PLAGA-Pluronic-PLAGA nanofibers containing 50 and 25% of glycolic acid units, respectively.

Considering the limited amount of BSA released after 50 days, we formulated nanofibers with additional porogen agents. Hydrophilic and biocompatible polymers with a similar molecular weight were chosen: Pluronic F127 (12,500 Da) at 29% *w*/*w* and PEG (10,000 Da) at 14% *w*/*w* in the electrospun solution. Other porogen concentrations or even other porogens (e.g., 4600 Da PEG) were tested without positive results. Therefore, only the optimized formulations will be discussed.

Figure 1d shows the comparative release from original nanofibers versus nanofibers containing porogen agents. The later series showed higher release efficiency of BSA. The addition of PEG increased the release efficiency of BSA from 21% to 34% for PLA50-Pluronic-PLA50 nanofibers. The same trend was also observed with the addition of Pluronic, with release efficiencies increased from 14% to 21% and from 27% to 49% for PLA_50_ and PCL nanofibers, respectively.

Following this first study of BSA release, two formulations have been selected for the inclusion of the growth factors IGF and HGF, namely PCL + Pluronic and PLA-Pluronic-PLA + PEG.

As an important factor that influences protein diffusion from nanofibers, the swelling ratios of the nanofibers were evaluated (Figure 1e). The maximum swelling was reached within 5 min for both polymers: PCL + Pluronic reached 621% of the swelling rate and PLA-Pluronic-PLA + PEG reached half of it, i.e., 295%. Despite this high water uptake, the mass loss of the nanofibers after 40 days of in vitro degradation was low and similar for both polymers because the remaining mass was 71 and 72% for PLA-Pluronic-PLA + PEG and PCL + Pluronic, respectively (Figure 1f). This implies that nanofiber degradation was not the cause of the difference in release kinetics between the different polymeric nanofibers during the first 40 days.

### 3.3. GF Loading and Release from Nanofibers

#### 3.3.1. GF Loading

**Figure 2:**
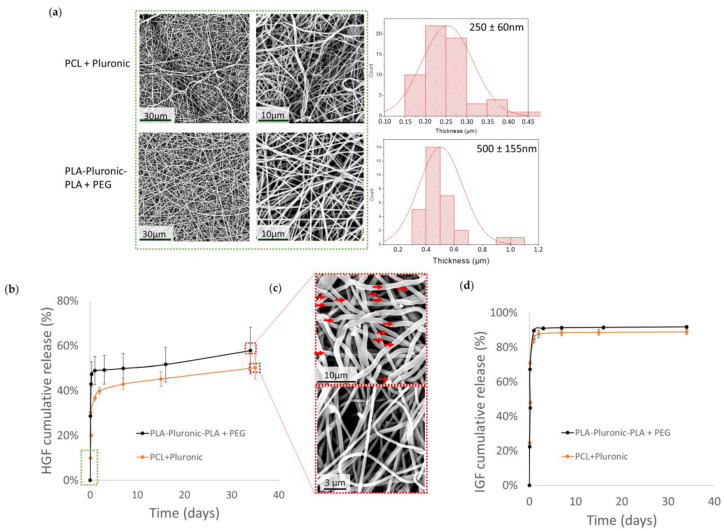
Growth factor loading and release kinetics from nanofibers: (**a**) SEM images and diameter distributions of HGF- and IGF-loaded nanofibers before release; (**b**) HGF release profile in PBS at 37 °C (scaffolds were loaded with 500 ng of IGF and 500 ng of HGF); (**c**) SEM images after 35 days of release in vitro (red arrows point to pores in the nanofibers); (**d**) IGF release profile in PBS at 37 °C (scaffolds were loaded with 1000 ng of IGF).

HGF and IGF were encapsulated in PCL + Pluronic and PLA-Pluronic-PLA + PEG nanofibers. The surface morphology of the nanofibrous scaffolds was examined using SEM. As shown in Figure 2a, both scaffolds showed a dense, compact, and uniform network of nanofibers, with average fiber diameters of 250 ± 60 nm and 500 ± 155 nm for PCL + Pluronic and PLA-Pluronic-PLA + PEG scaffolds, respectively.

#### 3.3.2. GF Release Kinetics

HGF release (Figure 2b) from PLA-Pluronic-PLA + PEG nanofibers started with a burst release of 47% and was then slowly stabilized to reach 58% after 35 days. The burst release from PCL + Pluronic nanofibers was lower (29%) and was also followed by a slow release, reaching 50% of the initial HGF loading after 35 days. SEM images (Figure 2c) of the scaffolds after 35 days of release in PBS at 37 °C showed the appearance of cracks (red arrows) due to PLA-Pluronic-PLA degradation.

Figure 2c shows that IGF was released with an important burst from PLA-Pluronic-PLA + PEG and PCL + Pluronic nanofibers. After two days, IGF release reached a plateau at 89% release efficiency from PLA-Pluronic-PLA + PEG nanofibers and 92% for PCL + Pluronic nanofibers.

#### 3.3.3. Impact of Association Methods on Release Kinetics

**Figure 3:**
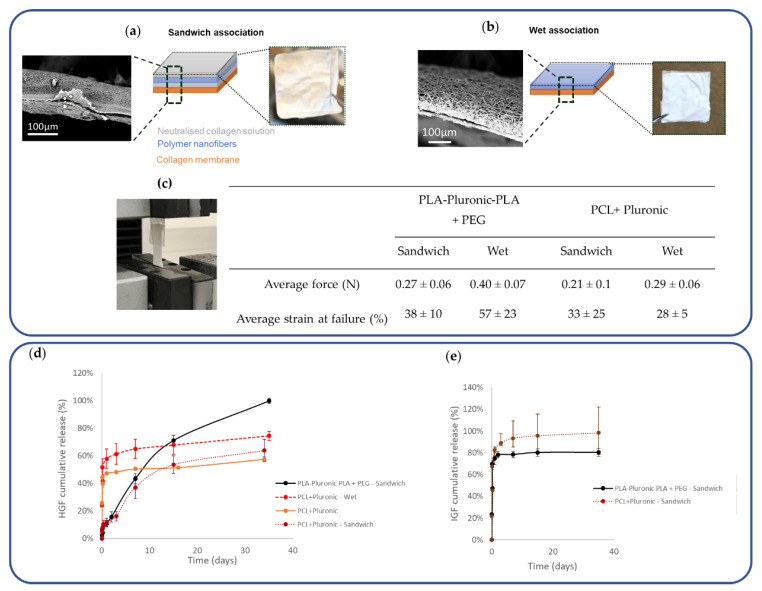
Impact of the method of association used to prepare the nanofiber–collagen membrane GF-eluting patches: (**a**) Schematic description of “sandwich association” and (**b**) “wet association”; (**c**) Lap-shear tests to determine the force required to separate the nanofiber–collagen interface; (**d**) Impact of association methods on HGF release kinetics; (**e**) Impact of association methods on IGF release kinetics.

With the aim of this study being to develop collagen-based patches with GF-eluting properties suitable for cardiac regeneration, we have evaluated two methods of association of the loaded nanofibers with the collagen membrane. In the first method, named “sandwich association”, the nanofiber mat was embedded in a collagen sponge that adhered to the collagen membrane (Figure 3a). In the second method, named “wet association”, a simple contact of the two materials after moistening was used (Figure 3b). The two components adhered together, as shown in the SEM images, and were manipulable in hydrated media. The force required to detach the nanofibers from the collagen membrane was the same (~0.25 N), regardless of the association mode and the composition of the nanofibers (Figure 3d).

The impact of the collagen association mode on HGF release was studied (Figure 3e). We observed an increase in HGF release efficiency from the PCL + Pluronic-Collagen patches obtained by wet association compared to the nanofibrous scaffolds alone, with the release increasing from 50% to 74% over the same period of 35 days. However, this mode of association further accentuated the burst phase, which reached 58% in 1 day. On the contrary, the “sandwich method”, which integrated the nanofibrous mat in freeze-dried collagen, reduced the burst to 12% in 1 day and yielded a more controlled and steady release over 35 days to reach 64% release efficiency. The same trend was observed for the patches made from PLA-Pluronic-PLA + PEG-Collagen, with a release efficiency of 100% after 35 days. On the other hand, the combination of nanofibers with collagen had no impact on the release of IGF, which was released in a large initial burst (67%) for both polymers, reaching 80% and 98% of the release in 24 h from the PLA-Pluronic-PLA + PEG collagen sandwich and the PCL + Pluronic collagen sandwich.

### 3.4. Cytocompatibility

**Figure 4:**
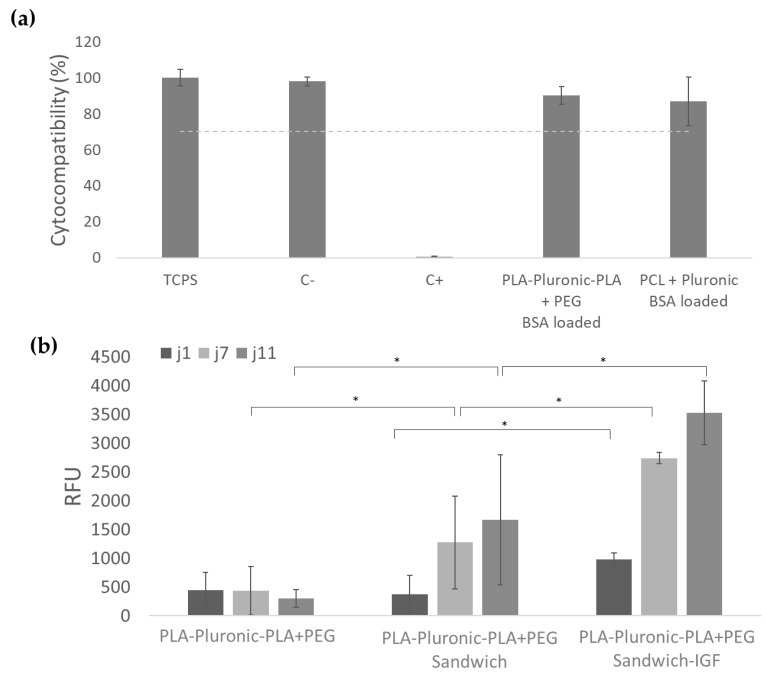
Biological compatibility of samples: (**a**) PLA-Pluronic-PLA + PEG BSA-loaded nanofibers and PCL + Pluronic BSA-loaded nanofibers were not cytotoxic after 72 h of contact with L929 cells compared to the negative control (high density polyethylene film), positive control (polyurethane film + 0.1% zinc diethyldithiocarbamate), and TCPS (Tissue culture polystyrene); (**b**) Myoblast C2C12 proliferation on PLA-Pluronic-PLA + PEG nanofibers, PLA-Pluronic-PLA + PEG sandwich patches, and PLA-Pluronic-PLA + PEG sandwich + IGF patches, * *p* < 0.05.

We wanted to verify that nanofibers were biocompatible. In view of the above results, we chose to focus on PLA-Pluronic-PLA + PEG patches that allow rapid release of IGF and sustained release of HGF in higher quantities than PCL + Pluronic patches. The percentage of viability of L929 in contact with PLA-Pluronic-PLA + PEG and PCL + Pluronic BSA-loaded nanofibers was 90% and 87% of the viability obtained on high density polyethylene film (negative control), which is higher than the 70% recommended by the European standard ISO 109993 (Figure 4a). This result shows that the nanofibers obtained were non-cytotoxic and can be used for cell-contacting biomedical applications. We also evaluated the myoblast C2C12 proliferation on different scaffolds (Figure 4b). The association of nanofibers with collagen membrane significantly improves cell proliferation after 7 and 11 days. The incorporation of IGF in the nanofibers also significantly impacts cell proliferation from the first day of culture.

## 4. Discussion

The objective of this work was to produce collagen-based patches with GF-eluting properties for at least 3 weeks that are suitable for cardiac regeneration. For this, we chose to encapsulate the growth factors in polymer nanofibers. Polymers were selected based on the criteria of compatibility with the electrospinning process, ease of processability, biocompatibility, degradability, and acceptability for biomedical applications. We focused specifically on the amorphous PLA_50_ (also referred to as PD,L-LA), which is a resorbable polyester classically used for drug delivery and that is FDA approved [22]. In order to modulate the diffusion of growth factors, we increased the hydrophilicity of PLA by preparing PLA copolymers by two means [23]. We first polymerized d,l-lactide from a Pluronic (F127) central block used as macro-initiator to yield PLA-Pluronic-PLA copolymers. In another approach, we also introduced glycolic units into the PLA blocks at different ratios to yield different types of PLGA-Pluronic-PLGA copolymers. Finally, we also used a commercial PCL whose high crystallinity and high hydrophobicity results in a much slower degradation rate than PLA [24]. The ring-opening polymerization of the various monomers has resulted in polymers and block copolymers with a molecular weight close to 100 kg/mol (see Table 1), suitable for electrospinning and molecule encapsulation.

As growth factors are very sensitive, BSA is commonly used as a carrier to protect them during the electrospinning process [21]. The protein has a molecular weight of 66 kDa and should therefore mainly regulate the diffusion of GF within the materials (7.6 kDa for IGF-1 and 79 kDa for HGF) [25]. We therefore first used BSA as a model protein by incorporating the protein into nanofibers and then carrying out a first diffusion study using nanofibers of different polymers. Despite the absence of BSA detected on the surface of the nanofibers, the release of BSA from the nanofibers was generally very rapid, but limited (15 to 36% as a function of the polymer; see Figure 1c). The introduction of Pluronic into the PLA backbone and then the introduction of glycolic units increased the burst and therefore improved the release efficiency of GF from polymeric nanofibers. However, between 64% and 85% of BSA still remained trapped in the nanofibers after 50 days. In order to promote the release of the trapped BSA, we added biocompatible porogens such as Pluronic, which is FDA approved and already entered in the composition of the copolymers (molecular weight 12.5 kg/mol) or PEG (molecular weight 10 kg/mol). These soluble macromolecules have the property to diffuse quickly from the nanofibers, creating pores that facilitate the diffusion of active ingredients in the nanofibers [26,27,28].

Due to their close molecular weight, few differences in BSA release kinetics were visible between the two porogens. However, the addition of porogens significantly reduced the amount of BSA still retained in the nanofibers after 35 days of release to values between 51 and 78%, compared to 64 and 85% without porogens, and thus increasing the release efficiency (Figure 1d). Correspondingly, increasing from 27 to 49%, the release efficiency of PCL nanofibers was improved by 22% with the addition of Pluronic (at polymer/porogen ratio 3:1). Similar results showed that the addition of Pluronic F127 into PLGA-Pluronic nanofibers improved the 10% porogen-free release efficiency to 25, 40, and 65% for polymer/porogen weight ratios of 5:1, 4:1, and 3:1, respectively [29]. In addition to pore creation, the pore-forming agent increases the hydrophilicity of the nanofibers, which in turn increases the absorption capacity of the aqueous medium and thus facilitates the diffusion of the BSA [29,30]. Similarly, the release efficiency of PLA-Pluronic-PLA nanofibers was improved by 15% with the addition of PEG (at polymer/porogen ratio 2:1). For comparison, it was shown in the literature that PEG (3.35 kg/mol) introduced at the same percentage as in our study improved the PCL release efficiency of SiRNA from 2.5% to 10% [28].

Finally, two polymers were selected for further work: PCL + Pluronic and PLA-Pluronic-PLA + PEG, which reached, respectively, 50 and 34% of BSA release after 42 days. We studied the release of growth factors from these two types of nanofibers. The release rates of HGF were similar for both polymers, with a burst phase followed by a sustained release that reached 50% for PCL + Pluronic and 58% for PLA-pluronic-PLA + PEG (Figure 2b). These results were not surprising because PCL is more hydrophobic than PLA. However, due to the high swelling rate (due to the higher content of Pluronic porogen at 29 wt%) and smaller diameter (250 ± 60 nm) of PCL + Pluronic nanofibers, the difference brought by hydrophobicity may have been lessened. The release of IGF from the nanofibers occurred in a large burst for both polymers in contrast to HGF release, which was more sustained. This can be explained by the molecular weight of HGF (79 kDa), which is 10 times higher than that of IGF (7.6 kDa), higher than that of BSA (66 kDA), and that leads to lower diffusion of HGF within the materials [31,32].

Although synthetic polymeric nanofiber scaffolds present great advantages owing to their good mechanical properties, excellent structural strength, and biodegradability, polymer nanofibers are not very favorable to cell adhesion. This is why polymer nanofibers have been associated in this work with a biomimetic material: collagen. Collagen alone presents relatively poor mechanical properties and is difficult to electrospin due to significant denaturation and degradation during electrospinning [33]. Chemical crosslinking can improve collagen strength and biodegradation rate, but this will inevitably lead to a sacrifice of chemical structure of the collagen, resulting, for example, to some loss of biocompatibility [34]. The association of the synthetic polymeric nanofibers and collagen should provide their respective advantages in a composite material.

We first associated a hydrated collagen membrane with HGF- and IGF-loaded nanofibers by simply placing the two materials in contact with each other and drying (Figure 3b). This simple association allowed the adhesion of the two materials but affected the release profile by increasing the burst of HGF. This important burst could be explained by an early release of growth factor and porogens during the association process due to the hydration of the membrane, which could at the same time result in channel creation due to porogens migration [26,27,28]. We therefore tested a second method of association by impregnating the two materials in a collagen solution and then freeze-drying the whole scaffold. This combination, called “sandwich”, also results in an easy to handle and cohesive patch (Figure 3a). Although we expected that impregnation in a collagen solution would increase the cohesion at the interface, this was not the case, as shown by the tensile values required to separate the two materials, which were similar regardless of the mode of association (Figure 3c). However, we observed the effect of the addition of a collagen sponge on the release profiles. Indeed, sandwich association, which integrated the nanofibrous layer in freeze-dried collagen, reduced the burst after 1 day from 40% to 12%. Thereafter, the release of HGF was much more sustained than without the presence of collagen and reached 64% in 35 days (Figure 3d). Similar to the wet scaffold, during scaffold fabrication, impregnation in the collagen solution could promote the diffusion of BSA and growth factors out of the nanofibers. However, unlike the wet scaffold, here the scaffolds were contained in a collagen gel that was subsequently freeze-dried. The release curves showed that after a slight burst, the GF release was delayed. This shift may be due to retention of the GFs by electrostatic binding of the collagen sponge and then release by desorption or degradation of the lyophilized collagen. The prolonged release achieved with this type of porous collagen matrix is described in the literature and led to significant GF retention. Some studies explain it by the ability of collagen to induce electrostatic interactions with GFs [35], while others advocate the mechanical effect of the material, namely the passage through an additional diffusion barrier that is thicker [36]. Finally, this delay generated the sequential release of the two GFs, which has already been described in the literature. Indeed, after encapsulation of IGF and HGF in alginate microparticles, 100% IGF was released in 1.5 days, while 30% HGF was released after 7 days [37]. Sequential release of IGF and HGF is of interest for myocardial regeneration after infarction. The rapid release of IGF-1 could provide an immediate signal for cell survival to reduce cell loss by apoptosis due to infarction and thus safeguard the remaining functional myocardium [38]. The slower and continuous release of HGF is active in the later phases of infarct repair, such as induction of angiogenesis, remodeling of the ECM, and reduction of fibrosis [39,40].

Previous works have shown that IGF, as well as HGF, administered in bolus are bioactive from 10 ng/mL [37]. Other studies show that both growth factors released from alginate nanoparticles were effective at doses ranging from 250 ng to 1000 ng and that their bioactivity increases with dose [11]. The bioactivity on C2C12 cells was evaluated with samples loaded with 1000 ng of IGF. As 80% of the IGF would be released within 24 h, the concentration of IGF available to the cells was 800 ng/mL, which allowed the promotion of cell proliferation, even after 11 days, when the medium was renewed (Figure 4b). This maintained bioactivity and positive impact of myoblasts proliferation confirm the suitability of the designed patches for cardiac applications.

## 5. Conclusions

The objective of this study was to design cardiac regeneration patches that release growth factors for 3 weeks. In order to prolong the release of GFs, we encapsulated them in PLA- or PCL-based nanofibers. To this aim, various PLA copolymers were synthesized and evaluated with respect to the ability of the nanofibers to control GF release. Beside composition, the addition of macromolecular porogens in nanofibers was also investigated to improve the efficiency of release. This study led to the selection of nanofibers composed of PCL + Pluronic and PLA_50_-Pluronic-PLA_50_ + PEG. To improve the biocompatibility of the scaffolds, we then tested two modes of association of the growth factor-loaded nanofibers with a collagen membrane. We demonstrated that a hybrid patch associated with a collagen membrane through a collagen sponge allowed the sequential release of GF by delaying and then retaining the release of HGF over 35 days, while the release of IGF was rapid. The combination of collagen and growth factors improved the bioactivity on C2C12, making such multi-GFs releasing patches promising for cardiac regeneration.

## Figures and Tables

**Table 1:** Characterization of polymers. ^1^H-NMR and SEC.

Polymers Composition	^1^H-NMR M*n* (kg·mol^−1^)	SEC	
		M*n* (kg·mol^−1^)	Ð
PLA50	124	113	2
PLA50-Pluronic-PLA50	97	68	1.6
PLA37.5GA25-Pluronic- PLA37.5GA25	99	65	1.5
PLA25GA50-Pluronic- PLA25GA50	101	67	1.4
PCL	80 ^1^	105	1.6

^1^ Theorical M*n* given by the supplier

## Data Availability

The data presented in this study are available on request from the corresponding author.

## Supplementary Materials

The following supporting information can be downloaded at: https://www.mdpi.com/article/10.3390/pharmaceutics14091854/s1: Table S1: Amount of monomers, initiator, and catalyst used; Table S2: Electrospinning parameters of BSA-loaded nanofibers; Table S3: Electrospinning parameters of GF-loaded nanofibers; Figure S1: ^1^ H-NMR spectra of (A) PLA50-PluronicF127-PLA50, (B) PLA37.5GA25-PluronicF127-PLA37.5GA25, and (C) PLA25GA50-PluronicF127-PLA25GA50 (300 MHz, CDCl_3_).
