# Supplementary material for: Design of Hybrid Polymer Nanofiber/Collagen Patches Releasing IGF and HGF to Promote Cardiac Regeneration
Source: Pharmaceutics. 2022 Sep 2;14(9):1854. doi: 10.3390/pharmaceutics14091854 (PMC9502465; doi:10.3390/pharmaceutics14091854)
Supplement: Supplementary file 1 [file pharmaceutics-14-01854-s001.zip › pharmaceutics-1851594-supplementary.pdf]

pharmaceutics

Table S1. Amount of monomers, initiator and catalyst used

	**PLA$_{50}$-Pluronic-PLA$_{50}$**	**PLA$_{37.5}$GA$_{25}$-Pluronic-PLA$_{37.5}$GA$_{25}$**	**PLA$_{25}$GA$_{50}$-Pluronic-PLA$_{25}$GA$_{50}$**
Mass of Lactide (g)	43.7	34.45	24.2
Mass of Glycolid (g)	-	9.25	19.50
Mass of pluronic (g)	6.3	6.3	6.3
Mass of catalyst (mg)	41	41	41

Table S2: Electrospinning parameters of BSA loaded nanofibers

Polymers	Electrospinning parameters<br>BSA loading<br>(polymers concentration, flow rate, needle- collector distance)
PLA$_{50}$	26%-0.6mL/h – 12kV-15cm
PLA$_{50}$-PluronicF127- PLA$_{50}$	30%-0.6mL/h – 12kV-15cm
PLA$_{37.5}$GA$_{25}$-PluronicF127-PLA$_{37.5}$GA$_{25}$	30%-0.6mL/h – 12kV-15cm
PLA$_{25}$GA$_{50}$-PluronicF127-PLA$_{25}$GA$_{50}$	25%-0.6mL/h – 12kV-15cm
PCL80k	17%-0.55mL/h – 14kV-15cm
PCL80k +PluronicF127(5%)	17%-0.65mL/h – 12kV-15cm
PLA$_{50}$ +PluronicF127(5%)	26%-0.9mL/h – 12kV-15cm
PLA$_{50}$-PluronicF127- PLA$_{50}$ +PEG10k	30%-0.9mL/h – 11kV-15cm
PLA$_{37.5}$GA$_{25}$-PluronicF127-PLA$_{37.5}$GA$_{25}$ + PEG10k	30%-0.75mL/h – 11kV-15cm

Table S3: Electrospinning parameters of GF loaded nanofibers

	PLA-Pluronic-PLA+PEG +GF	PCL+Pluronic +GF
Polymers Concentration	22.5%	12.75%
Mass ratio porogen / Polymers	19%	39%
Electrospinning parameters	0,2mL / 0,8mL/h / 12kV / 15cm	0,2mL / 0,65mL/h / 12kV / 15cm

Figure S1. **[1] H-NMR spectra of (A) PLA50-PluronicF127-PLA50, (B) PLA37.5GA25-PluronicF127-PLA37.5GA25 and (C) PLA25GA50-PluronicF127-PLA25GA50 (300 MHz, $CDCl_3$).**

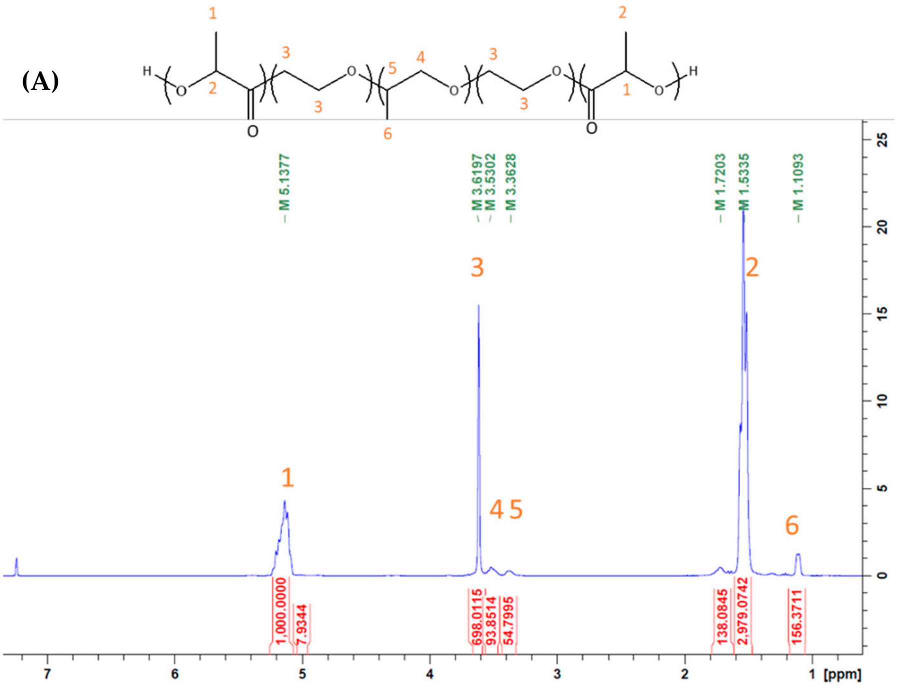

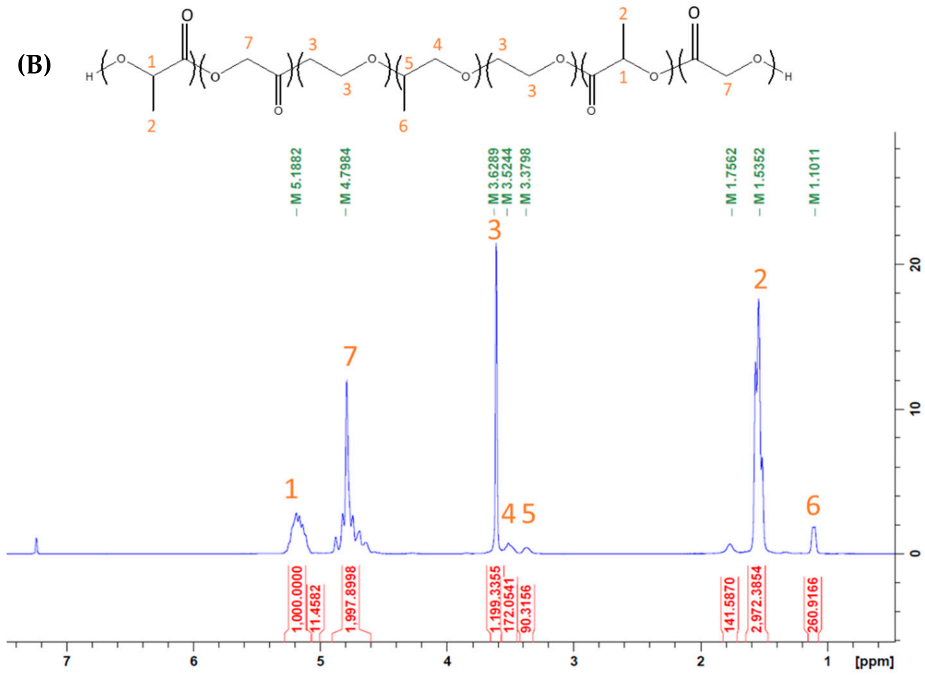
(B)
M 5.1882
M 4.7984
M 3.6289
M 3.5244
M 3.3798
M 1.7562
M 1.5352
M 1.1011
1.000.0000
11.4582
1.997.8998
1.199.3355
172.0541
90.3156
141.5870
2.972.3854
260.9166
[ppm]

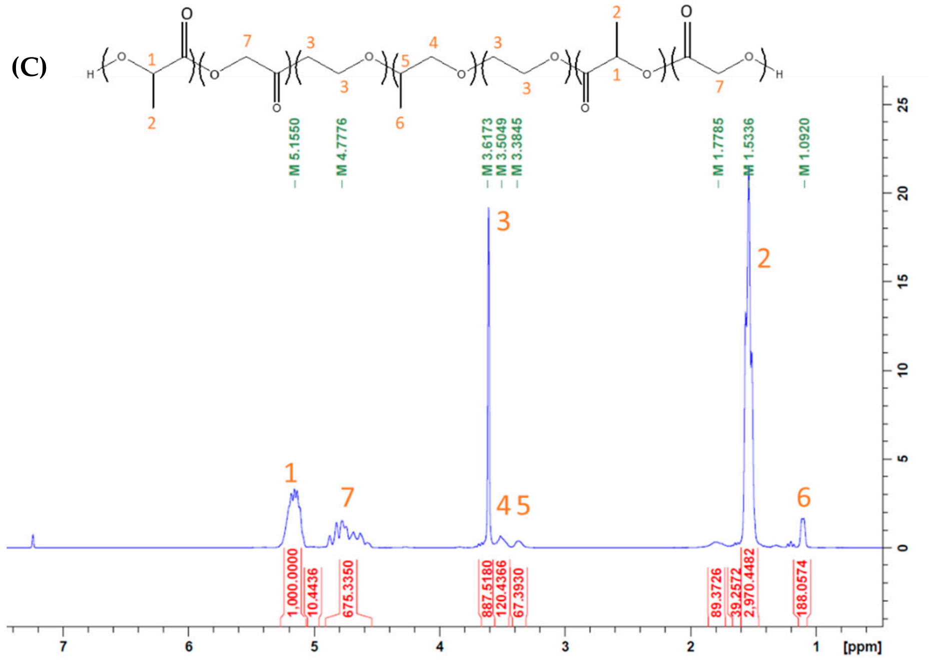
(C)
M 5.1550
M 4.7776
M 3.6173
M 3.5049
M 3.3845
M 1.7785
M 1.5336
M 1.0920
1.000.0000
10.4436
675.3350
887.5180
120.4366
67.3930
89.3726
39.2572
2.970.4482
188.0574
[ppm]
